# Patient satisfaction survey of a newly established footwear bank in a rehabilitation hospital

**DOI:** 10.1186/1757-1146-4-S1-P10

**Published:** 2011-05-20

**Authors:** Christine Corkhill, Adrian Eastham

**Affiliations:** 1 Department of Podiatry, Eastern Health, Australia

## Background

The Peter James Centre (PJC) is the main rehabilitation hospital in Eastern Health. It provides a comprehensive inpatient and community rehabilitation programme. A Footwear Bank was established by the Podiatry Department in 2009 following an audit in 2007 that found 30% of the patients were not wearing suitable shoes to their therapy. The problem of finding suitable footwear had been made more difficult because three of the local shoe shops closed down. The Footwear Bank stocks several types of shoes, all with Velcro straps. The three objectives were to assess patient satisfaction regarding the service of the Footwear Bank, the quality of the footwear and whether the footwear was still being worn after discharge from PJC.

## Methods

Patients who had purchased footwear were contacted by telephone for a five question survey. Their responses were collated and analysed in an Excel spreadsheet. The results were separated into groups depending on the shoe, to determine if there were any issues associated with a particular shoe.

## Results

Twenty-eight of the fifty-five patients who had purchased footwear were able to be contacted for the questionnaire. Of these, 22 (79%) considered the shoes to be successful; 5 (18%) considered them somewhat successful; 1 (3%) did not consider them successful, though still wore them some of the time. Eighteen (64%) did not have any troubles with the shoes. Twenty-six (93%) are currently still wearing the shoes; 10 of the 28 (36%) wore the shoes all the time; 6 (21%) wore them most of the time; 10 (36%) wore them some of the time; 2 (7%) never wore them. Twenty-six of the 28 (93%) patients were happy with the service.

## Conclusion

The Footwear Bank was established in response to patients’ needs and aimed to improve health. While this survey is small in size and does have limitations, the responses overall were positive and indicate that it has been successful. There were no reports of shoes wearing out or physical faults developing from wear and tear, indicating that the shoes are of good quality.

